# Pharmacological inhibition of PI3K class III enhances the production of pro- and anti-inflammatory cytokines in dendritic cells stimulated by TLR agonists

**DOI:** 10.1016/j.intimp.2016.04.028

**Published:** 2016-07

**Authors:** Álvaro Pittini, Cecilia Casaravilla, Judith E. Allen, Álvaro Díaz

**Affiliations:** a Cátedra de Inmunología, Departamento de Biociencias (Facultad de Química) e Instituto de Química Biológica (Facultad de Ciencias), Universidad de la República, Montevideo, Uruguay; b Institute of Immunology and Infection Research Centre for Immunity, Infection and Evolution, School of Biological Sciences, University of Edinburgh, Edinburgh, UK

**Keywords:** Wortmannin, PI3K, Vps34, Dendritic cells, IL-10

## Abstract

The phosphatidylinositol 3-kinase (PI3K) pathway is known to down-regulate inflammatory cytokine responses in dendritic cells and macrophages stimulated with TLR agonists. This is due to class I PI3Ks causing the activation of Akt, which in turn inactivates GSK3, a kinase that promotes the transcription of IL-12 and represses that of anti-inflammatory IL-10. Using bone marrow-derived dendritic cells we find that whereas pharmacological inhibition of Akt or GSK3 has the expected effects on these cytokines, the widely used PI3K inhibitor wortmannin causes a paradoxical increase in the production of IL-10. Wortmannin inhibits all PI3K classes, including PI3K class III, involved in endosomal function and autophagy, for which specific inhibitors were until recently not available. Using inhibitors specific for PI3K class III *vs* class I, we show that whereas inhibition of class I PI3K has the expected opposing effects on IL-10 and IL-12 production, inhibition of class III PI3K enhances the production of both of these, plus further cytokines. This explains the paradoxical inhibition of IL-10 production by wortmannin.

## Highlights

• The PI3K-Akt pathway is anti-inflammatory in myeloid cells.
• Its activation during TLR stimulation up-regulates anti-inflammatory IL-10.
• The general PI3K inhibitor wortmannin has the opposite effect to expected on IL-10.
• Inhibitors specific for PI3Ks of classes I and III explain this paradox.
• Inhibition of PI3K class III enhances the production of IL-10 and other cytokines.

## 1 Introduction

The phosphoinositide 3-kinase (PI3K) enzyme family is involved in several central aspects of cell and tissue biology, including cell survival and proliferation, metabolism, autophagy, and inflammation. All PI3Ks are composed of a C2 domain, a helical domain, and a catalytic domain [1]. The PI3K classification depends on the presence of additional protein domains, their interactions with regulatory subunits, and the 3-phosphorylated phosphoinositides that they synthesise. Class I PI3Ks are formed by four different catalytic subunit isoforms, namely PI3Kα, PI3Kβ, PI3Kγ and PI3Kδ, which heterodimerise with different regulatory subunits. There are three isoforms of class II PI3K, namely PI3KC2α, PI3KC2β and PI3KC2γ. Lastly, there is only one catalytic subunit of class III PI3K called VPS34 (vacuolar protein sorting 34).

Class I PI3Ks generate phosphatidylinositol (3,4,5)-triphosphate, PI(3,4,5)P_3_ [1]. One of the main effectors of class I PI3Ks is the kinase Akt (PKB). Akt is recruited to membranes by PI(3,4,5)P_3_, where it is phosphorylated by PDK-1 (on T308) and mTORC2 (on S473) for its complete activation [2]. This PI3K/Akt pathway regulates cell survival, translation, metabolism, and immune responses [1]. An important molecular target of this pathway is glycogen synthase kinase 3 (GSK3), which is phosphorylated and inactivated by Akt [3], [4]. The PI3K/Akt/GSK3 “sub-pathway” so formed (Supplementary Fig. 1) has an important role in innate immunity. Specifically, it is well known to down-regulate the pro-inflammatory cytokine IL-12 and to up-regulate the anti-inflammatory cytokine IL-10 in myeloid cells stimulated with TLR agonists [5], [6], [7], [8], [9], [10], [11], [12]. TLR stimulation is accompanied by PI3K and Akt activation and therefore inactivation of GSK3, the activity of which influences the expression of IL-12 in a positive way and that of IL-10 in a negative way. In this context, Akt has been shown to inactivated GSK3 both directly as mentioned above, and indirectly through P70S6 kinase (P70S6K); P70S6K is activated by the mTOR complex 1, in turn activated downstream of PI3K and Akt [3], [11].

In short, through inactivating GSK3, the PI3K/Akt pathway prevents excessive inflammatory responses after TLR activation. For the capacity of the pathway to downregulate IL-12, pharmacological evidence agrees with the evidence generated from gene-targeted mice [5], [8], [9], [13], [14], [15]. This includes evidenced obtained with wortmannin, the most widely used PI3K inhibitor, known to be free of the specificity problems affecting LY294002 in particular [12]. In contrast, for IL-10 upregulation, results obtained with wortmannin [16], [17], often clash with the evidence based on genetically modified mice [14], [15], [18], [19]. However, the results generated using a specific inhibitor of the catalytic subunit p110δ do agree with the data from genetically modified mice [14]. Thus, it seems likely that the effects of wortmannin on other targets, including non-class I PI3Ks, could explain these disagreements.

Class III PI3K, VPS34, generates phosphatidylinositol 3-phosphate, PI(3)P [1]. VPS34 is active as part of at least two complexes with different cellular localizations and roles [20]. Thus VPS34 regulates membrane trafficking, autophagy, and it is also proposed to participate in amino acid sensing upstream of mTORC1 activation [20], [21]. Whereas VPS34 is targeted by wortmannin and other pan-PI3K inhibitors such as 3-methyladenine, specific inhibitors for this kinase were described only in the last two years [22], [23], [24]. In this study, we make use of these new inhibitors to explore the impact of VPS34 inhibition on the cytokine responses of dendritic cells to TLR agonists. Our results help to explain the paradoxical effects of wortmannin on IL-10 production.

## 2 Materials and methods

### 2.1 Antibodies and reagents

Antibodies against Akt and phosphorylated Akt (S473) were purchased from Cell Signaling Technology. Antibody to α-tubulin was from Santa Cruz Biotechnology. Secondary antibodies, anti-IgG and anti-IgM, both HRP-conjugated, were from Calbiochem and Invitrogen, respectively. Wortmannin was purchased from Sigma, Akt inhibitor VIII (Akt VIII) from Merck-Millipore, and GDC-0941 and SB216763 from ApexBio. SAR405 and VPS34IN-1 were purchased from the Division of Signal Transduction Therapy (DSTT) Unit at the University of Dundee. LPS and Pam3CSK4 were purchased from Sigma and InvivoGen, respectively.

### 2.2 Generation of murine bone-marrow-derived dendritic cells (BMDCs)

BMDCs were obtained by the method of Lutz et al. [25] as described in detail in [26]. Recombinant mouse granulocyte-macrophage colony-stimulating factor (GM-CSF) was from PeproTech. All stimuli were added in medium containing 5 ng/mL GM-CSF.

### 2.3 Immunoblotting

Immunoblotting analysis was performed following standard procedures. BMDCs were lysed in PBS pH 7,2, 0.5% *w*/*v* Triton X-100 (Applichem), containing protease and phosphatase inhibitor cocktails from Santa Cruz Biotechnology. Lysates were resolved on SDS-PAGE and transferred onto polyvinylidene fluoride membranes from Merck-Millipore. Membranes were blocked in PBS, 0.1% *w*/*v* Tween 20 (Sigma) and 0.5% *w*/*v* BSA (Sigma), probed with the corresponding antibodies and developed with the SuperSignal™ West Pico Chemiluminescent Substrate (ThermoFisher).

### 2.4 Measurement of cytokines

BMDCs were treated with inhibitors 30 min before stimulation with TLR agonists. IL-10, IL-12p70, IL-6 and tumor necrosis factor alpha (TNF-α) were measured in cultured supernatants, after 18 h of BMDCs stimulation, using ELISA kits from BD Biosciences.

### 2.5 Statistical analyses

The intra-experiment statistical analyses were carried out by one-way analysis of variance (ANOVA), with a Tukey post-test. The inter-experiment statistics (*i.e.* putting together the results of repeated independent experiments) were carried out by the restricted maximum-likehood (REML) method [27], also with a Tukey post-test.

## 3 Results and discussion

### 3.1 Wortmannin causes a paradoxical increase in IL-10 production in BMDCs stimulated with TLR agonists

**Fig. 1:**
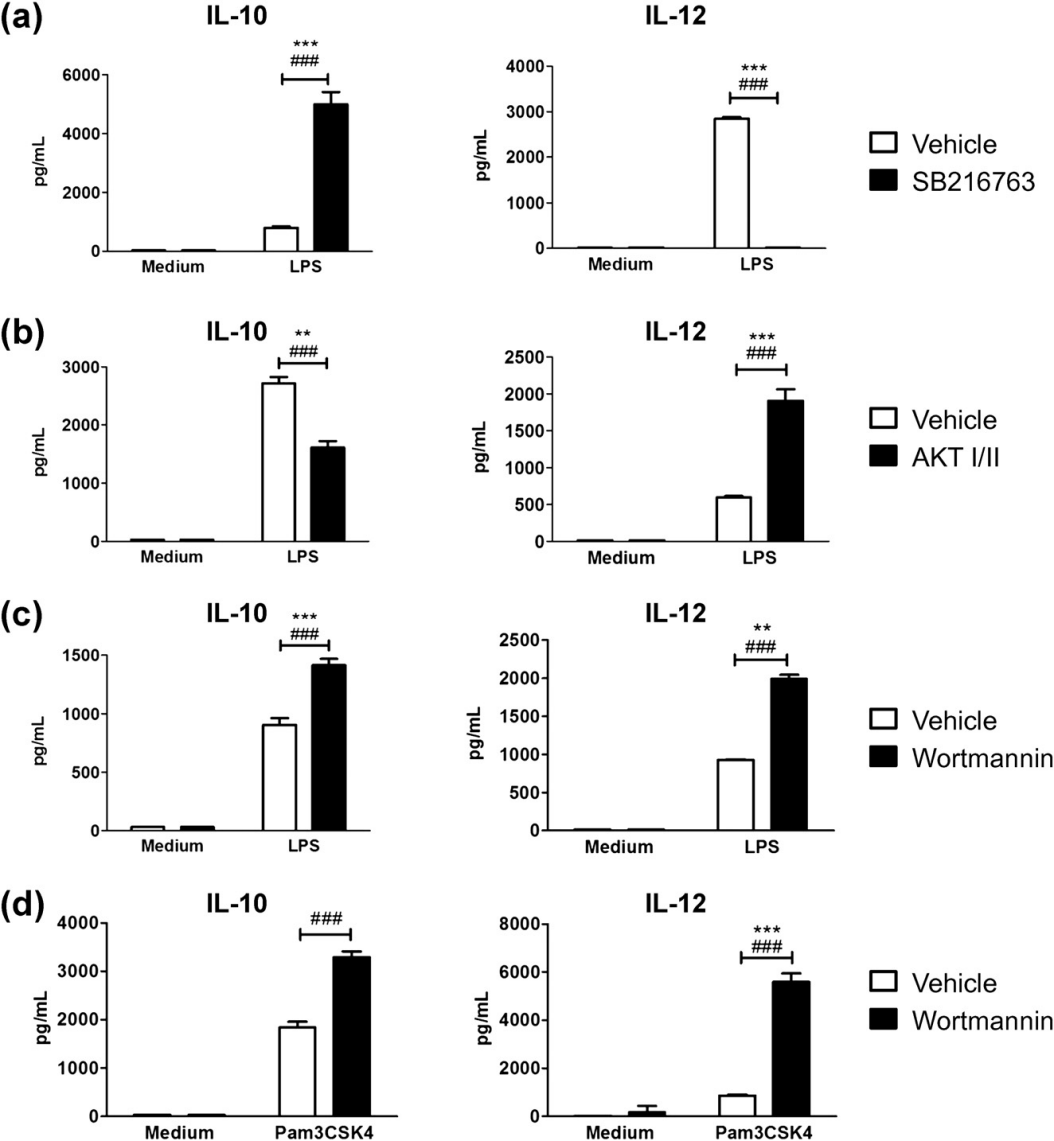
Wortmannin does not affect IL-10 production in TLR-stimulated BMDCs as expected from the anti-inflammatory nature of the PI3K-Akt-GSK3 sub-pathway. BMDCs were pretreated with inhibitors or vehicle (DMSO) for 30 min before stimulation with 10 ng/mL LPS (a–c) or 200 ng/mL Pam3CSK4 (d). Eighteen hours later, IL-10 and IL-12p70 were quantified by ELISA in supernatants. Inhibitors tested were SB216763 (10 μM, for GSK3) (a), Akt I/II (10 μM, for Akt) (b), and wortmannin (100 nM, for PI3K) (c, d). All data are presented as mean ± SD of triplicate wells. The results shown are representative of 3 independent experiments. Inter-experiment statistics are shown (***, P < 0.001; **, P < 0.01, * P < 0.05), along with intra-experiment ones (###, P < 0.001; ##, P < 0.01, # P < 0.05).

Because the PI3K/Akt/GSK-3 sub-pathway is known to regulate the production of IL-10 and IL-12 in response to TLR agonists in myeloid cells [5], [6], [7], [8], [9], [10], [11], we chose to study how the inhibition of each of these kinases affects the production of IL-10 and IL-12 in BMDCs stimulated with LPS (Fig. 1). As expected, a specific inhibitor of GSK-3 (SB216763) increased IL-10 production whereas it decreased IL-12 production (Fig. 1A). Also as expected, the inhibition of Akt (by Akt inhibitor VIII) decreased IL-10 production and increased IL-12 production (Fig. 1B). However, the inhibition of PI3Ks by wortmannin, while increasing IL-12 production as expected, did not decrease IL-10 production, and it actually increased it, both after stimulation with LPS and with the TLR2 agonist Pam3CSK4 (Fig. 1C and D). This is similar to the increase in IL-10 production induced by wortmannin reported previously in macrophages [19].

### 3.2 VPS34 inhibition enhances the production of both IL-10 and IL-12 in BMDCs stimulated with TLR agonists

**Fig. 2:**
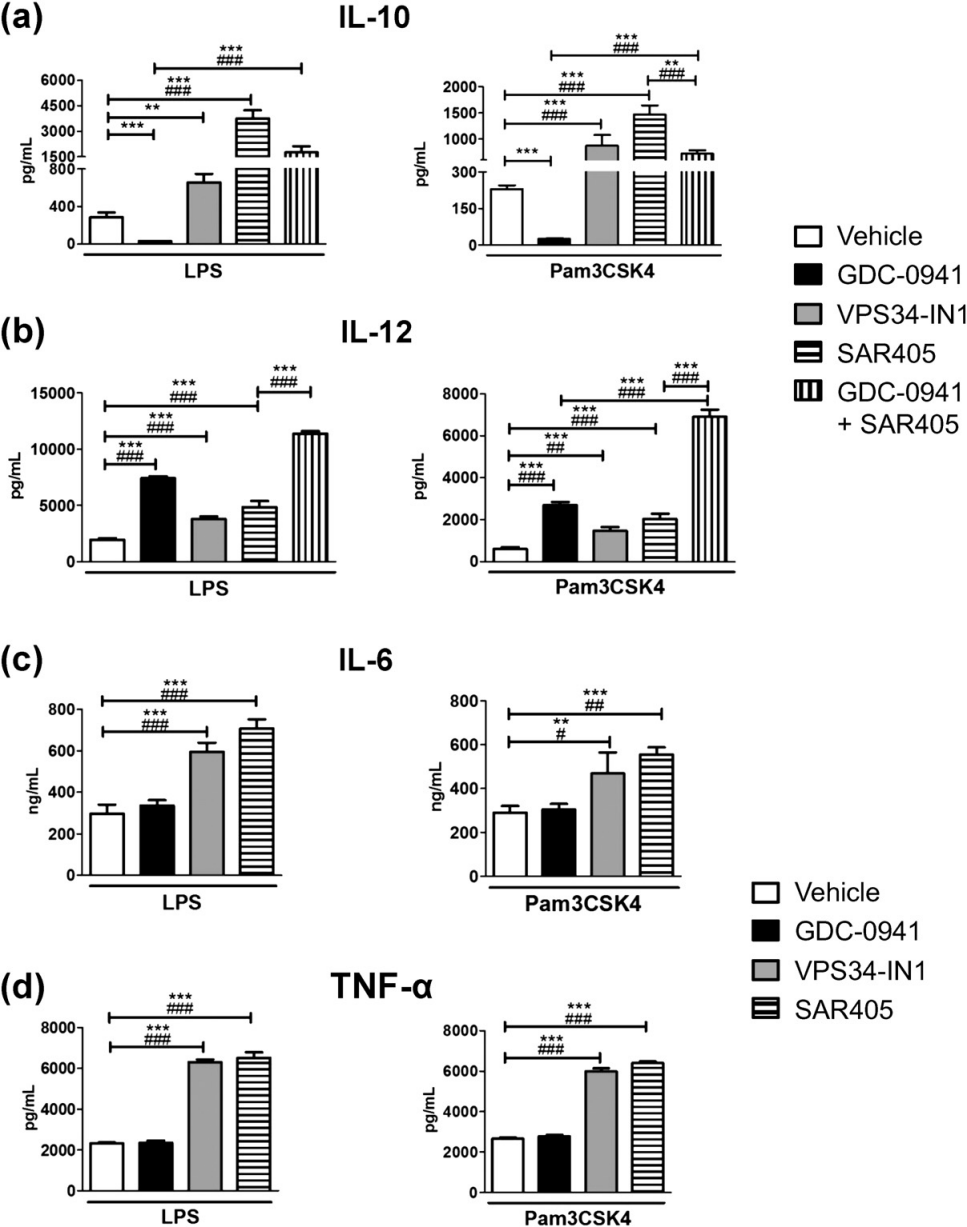
PI3K class I and class III inhibitors both enhance the production of proinflammatory cytokines in TLR-stimulated BMDCs, but they have opposite effects on the production of IL-10. BMDCs were pretreated with inhibitors or vehicle (DMSO) for 30 min before stimulation with 10 ng/mL LPS or 200 ng/mL Pam3CSK4 as indicated. Inhibitors tested were GDC-0941 (1 μM, for PI3K class I), VPS34-IN1 (1 μM, for PI3K class III), SAR405 (1 μM, for PI3K class III), or a mixture of GDC-0941 and SAR405 (1 μM each; only in parts (a) and (b)). Eighteen hours later, IL-10 (a), IL-12p70 (b), IL-6 (c) and TNF-α (d) were quantitated by ELISA in the supernatants. No significant levels of cytokines were detected in BMDCs incubated in media without TLRs agonist. All data of results are given as means ± SD of triplicate wells. Results are representative of 3 independent experiments. Statistical significances are expressed as for Fig. 1.

Since wortmannin is a pan-PI3K inhibitor, we speculated that the paradoxical increase in IL-10 production caused by this drug may be due to inhibition of VPS34. In order to investigate this issue, we used two structurally unrelated inhibitors of this kinase, namely SAR405 and VPS34-IN1 [22], [23]. We verified that the phosphorylation of Akt (S473) was abrogated by wortmannin, Akt inhibitor VIII and the PI3K class I-specific inhibitor GDC-0941, but not by the new VPS34 inhibitors (Supplementary Fig. 2). The VPS34 inhibitors had only a minor negative effect on Akt phosphorylation; this is unlikely to be a direct effect on PI3K class I, since it has been shown that neither inhibitor affects significantly the activity of PI3K class I at the concentration used in our experiments (1 μM) [22], [23]. In contrast to the PI3K class I-specific inhibitor (GDC-0941), which caused the expected decrease in IL-10 production, both VPS34 inhibitors increased IL-10 production in BMDCs stimulated with either LPS or Pam3SCK4 (Fig. 2A). Simultaneous inhibition of PI3Ks class I and III (by the combined use of GDC-0941 and SAR405) had as the net effect an enhancement in IL-10 production. In other words, the combination of specific PI3K class I and III inhibitors imitated the effect of wortmannin. Hence the paradoxical effect of wortmannin on IL-10 production is likely explained by the inhibition of VPS34, which has a negative effect on this cytokine.

We also evaluated whether the VPS34 inhibitors affect the production of IL-12. SAR405 and VPS34-IN1 increased IL-12 production, as did GDC-0941 (Fig. 2B). The effect of VPS34 inhibition was weaker than that of PI3K class I inhibition, a difference that may be at least partially explained by the enhanced production of IL-10, known to down-regulate IL-12 in an autocrine manner [8]. The combination of PI3K class I and class III inhibition induced a large increase in IL-12 production in response to LPS or to Pam3CSK4, suggesting an additive effect of both classes of PI3Ks on the production of this cytokine.

### 3.3 VPS34 inhibition enhances the production of further cytokines in BMDCs stimulated with TLRs agonists

Finally, we assessed whether the effects of VPS34 are specific to IL-10 and IL-12, or extend to further cytokines. For this purpose, we analyzed the production of TNF-α and IL-6 in BMDCs stimulated with LPS and Pam3CSK4, in the presence of the PI3K class-specific inhibitors (Fig. 2C and D). GDC-0941 did not affect the production of TNF-α or IL-6. This differed from the data obtained by Aksoy et al. [15] using BMDCs carrying a kinase-dead version of PI3Kδ, which suggests that different PI3K class I isoforms may influence TNF-α and IL-6 differently. More importantly, both PI3K class III inhibitors significantly increased the production of TNF-α and IL-6 elicited by either TLR agonist tested. We also analyzed the effects of the PI3K inhibitors on the secretion of the low levels of IL-1β elicited by TLR agonists in the absence of inflammasome activators (Supplementary Fig. 3). The VPS34 inhibitors, but not the class I-specific inhibitor, significantly increased the production of IL-1β induced by LPS; a similar enhancement had been previously reported in the presence of 3-methyladenine, which inhibits both PI3K class I and class III [28]. However, the potentiation of IL-1β output by VPS34 inhibitors was absent when Pam3CSK4 was used as a stimulus, suggesting that the situation for this cytokine is different than for conventionally secreted cytokines.

### 3.4 Concluding remarks

Taken together our results show that inhibition of VPS34 causes increases in the production of several conventionally secreted cytokines in BMDCs stimulated with TLR agonists. They also show that this enhancement, which affects both pro- and anti-inflammatory cytokines, becomes superimposed on the expected pro-inflammatory effects of inhibiting PI3K class I when an inhibitor targeting both PI3K class I and class III, such as wortmannin, is used. The mechanism underlying the observed effect of VPS34 inhibition is not obvious. VPS34 is necessary for TLR9 signaling, which starts in endosomes [29], but this cannot explain the enhancement of cytokine responses after VPS34 inhibition, nor explain effects in response to TLR family members (TLR2; for pro-inflammatory responses, TLR4) that signal from the cell surface. The mechanisms underlying our observation may well be complex, as VPS34 inhibition can be expected to have profound effects on the basic cellular functions of autophagy and vesicular trafficking [20]. When using 18 h or similarly long endpoints, as it is the case in our work and many others, such alteration in housekeeping cellular processes is likely to result in effects impacting on many cellular functions. Therefore our results do not imply necessarily that VPS34 specifically controls the cytokine output of dendritic cells under physiological conditions. However, they do imply that the use of pan-PI3K inhibitors to explore the functionality of the PI3K pathway carries the risk of a confounding general enhancement in the cytokine output of cells as a result of VPS34 inhibition.

## Conflict of interest

No conflict of interest declared.

## Appendix A Supplementary data

Suplementary Fig. 1. PI3K-Akt-GSK3 sub-pathway. Simplified representation of PI3K Akt/GSK3 “sub-pathway”. PI3K class I can be activated by various stimuli, including TLR agonists. Activated PI3K class I catalyses the phosphorylation of PI(4,5) P2 to form PI(3,4,5)P3 at the plasma membrane. PI(3,4,5) P3 recruits Akt to the plasma membrane, where it is phosphorylated by PDK1 and mTORC2 in the positions T308 and S473, respectively. Both phosphorylation events are necessary for the complete activation of Akt. Activated Akt directly phosphorylates GSK3α/β in the position S21 (GSKα) or S9 (GSKβ), inactivating GSK3. Also, Akt brings about the inactivation of GSK3 indirectly, through the activation of mTORC1 and consequently of P70S6K, which phosphorylates GSK3. In innate immune cells, GSK3 promotes the expression of IL-12 and restrains that of IL-10. Thus the PI3K/Akt-mediated inactivation of GSK3 upregulates IL-10 and down-regulates IL-12 production. Supplementary Fig. 2. Effects of the inhibitors used on Akt phosphorylation. BMDCs were pretreated with the inhibitors wortmannin (100 nM), Akt I/II (10 μM), GDC-0941 (1 μM), SAR405 (1 μM), VPS34-IN1 (1 μM), GDC-0941 + SAR405 (1 μM each), or with vehicle only (DMSO) for 30 min before stimulation with 10 ng/mL LPS. Eighty minutes later Akt phosphorylation (S473) was measured by Western blot, using α-tubulin as loading control. Results are representative of 2 independent experiments. Supplementary Fig. 3. Effects of VPS34 inhibition on the production of IL-1β by BMDCs stimulated with LPS or Pam3CSK. BMDCs were pretreated with GDC-0941 (1 μM), VPS34-IN1 (1 μM), SAR405 (1 μM) or only vehicle (DMSO) for 30 minutes before stimulation with 10 ng/mL LPS (a) or Pam3CSK4 (b). Eighteen hours later, IL-1β was quantitated in the supernatants by ELISA. No significant levels of the cytokine were detected in BMDCs incubated in media without TLRs agonist. All data are presented as mean ± SD of triplicate wells. Results are representative of 3 independent experiments. Statistical significances are given as for Fig. 1. Image 1
