# Supplementary material for: Pharmacological inhibition of PI3K class III enhances the production of pro- and anti-inflammatory cytokines in dendritic cells stimulated by TLR agonists
Source: Int Immunopharmacol. 2016 Jul;36:213–7. doi: 10.1016/j.intimp.2016.04.028 (PMC4907315; doi:10.1016/j.intimp.2016.04.028)
Supplement: Supplementary file 1 — Suplementary Fig. 1. PI3K-Akt-GSK3 sub-pathway. Simplified representation of PI3K Akt/GSK3 “sub-pathway”. PI3K class I can be activated by various stimuli, including TLR agonists. Activated PI3K class I catalyses the phosphorylation of PI(4,5) P2 to form PI(3,4,5)P3 at the plasma membrane. PI(3,4,5) P3 recruits Akt to the plasma membrane, where it is phosphorylated by PDK1 and mTORC2 in the positions T308 and S473, respectively. Both phosphorylation events are necessary for the complete activation of Akt. Activated Akt directly phosphorylates GSK3α/β in the position S21 (GSKα) or S9 (GSKβ), inactivating GSK3. Also, Akt brings about the inactivation of GSK3 indirectly, through the activation of mTORC1 and consequently of P70S6K, which phosphorylates GSK3. In innate immune cells, GSK3 promotes the expression of IL-12 and restrains that of IL-10. Thus the PI3K/Akt-mediated inactivation of GSK3 upregulates IL-10 and down-regulates IL-12 production. Supplementary Fig. 2. Effects of the inhibitors used on Akt phosphorylation. BMDCs were pretreated with the inhibitors wortmannin (100 nM), Akt I/II (10 μM), GDC-0941 (1 μM), SAR405 (1 μM), VPS34-IN1 (1 μM), GDC-0941 + SAR405 (1 μM each), or with vehicle only (DMSO) for 30 min before stimulation with 10 ng/mL LPS. Eighty minutes later Akt phosphorylation (S473) was measured by Western blot, using α-tubulin as loading control. Results are representative of 2 independent experiments. Supplementary Fig. 3. Effects of VPS34 inhibition on the production of IL-1β by BMDCs stimulated with LPS or Pam3CSK. BMDCs were pretreated with GDC-0941 (1 μM), VPS34-IN1 (1 μM), SAR405 (1 μM) or only vehicle (DMSO) for 30 minutes before stimulation with 10 ng/mL LPS (a) or Pam3CSK4 (b). Eighteen hours later, IL-1β was quantitated in the supernatants by ELISA. No significant levels of the cytokine were detected in BMDCs incubated in media without TLRs agonist. All data are presented as mean ± SD of triplicate wells. Results are representative of 3 [file mmc1.pptx]

## Slide 1
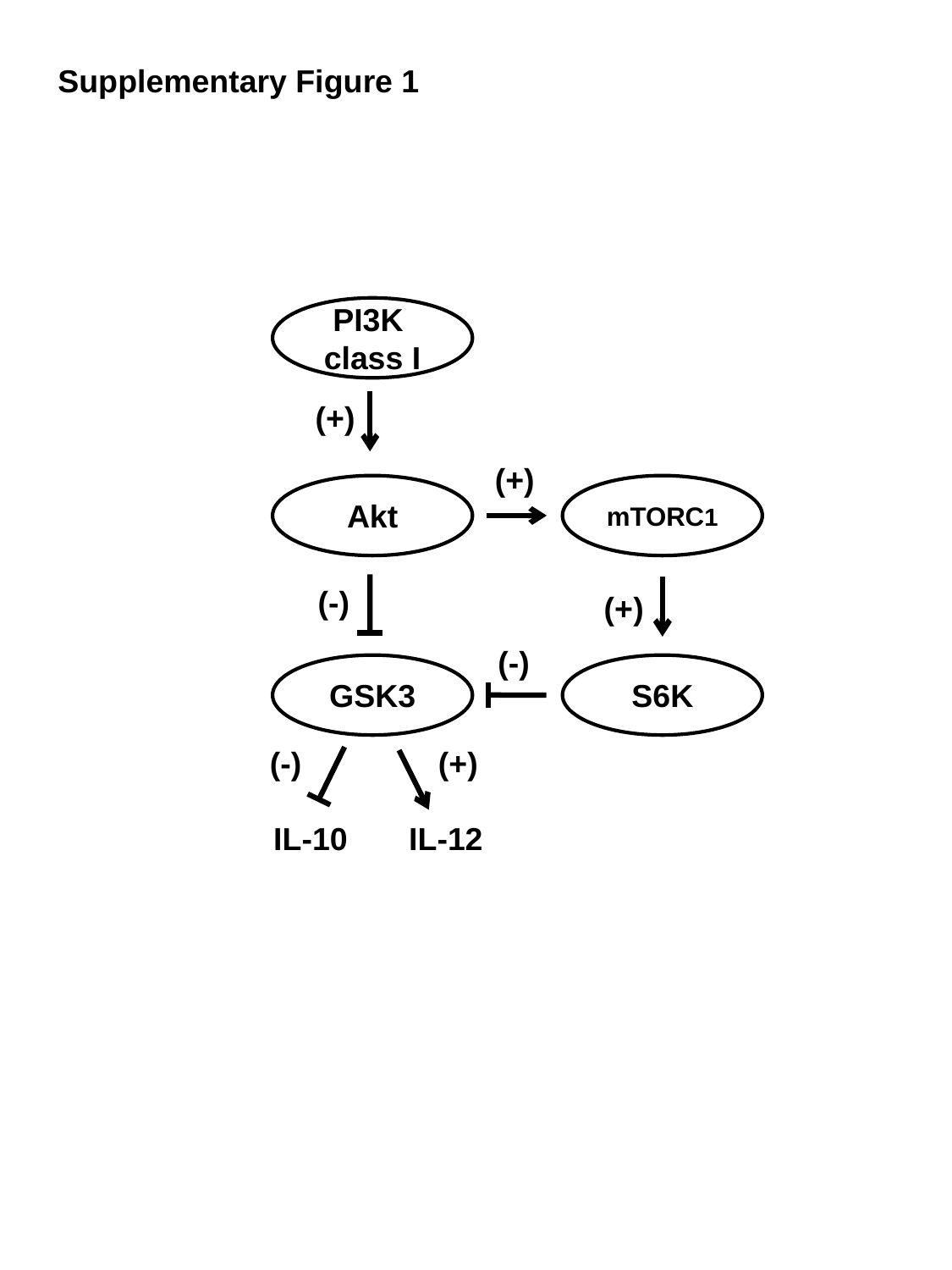

Supplementary Figure 1
PI3K
class I
(+)
(+)
Akt
mTORC1
(-)
(+)
(-)
GSK3
S6K
(-)
(+)
IL-10
IL-12

## Slide 2
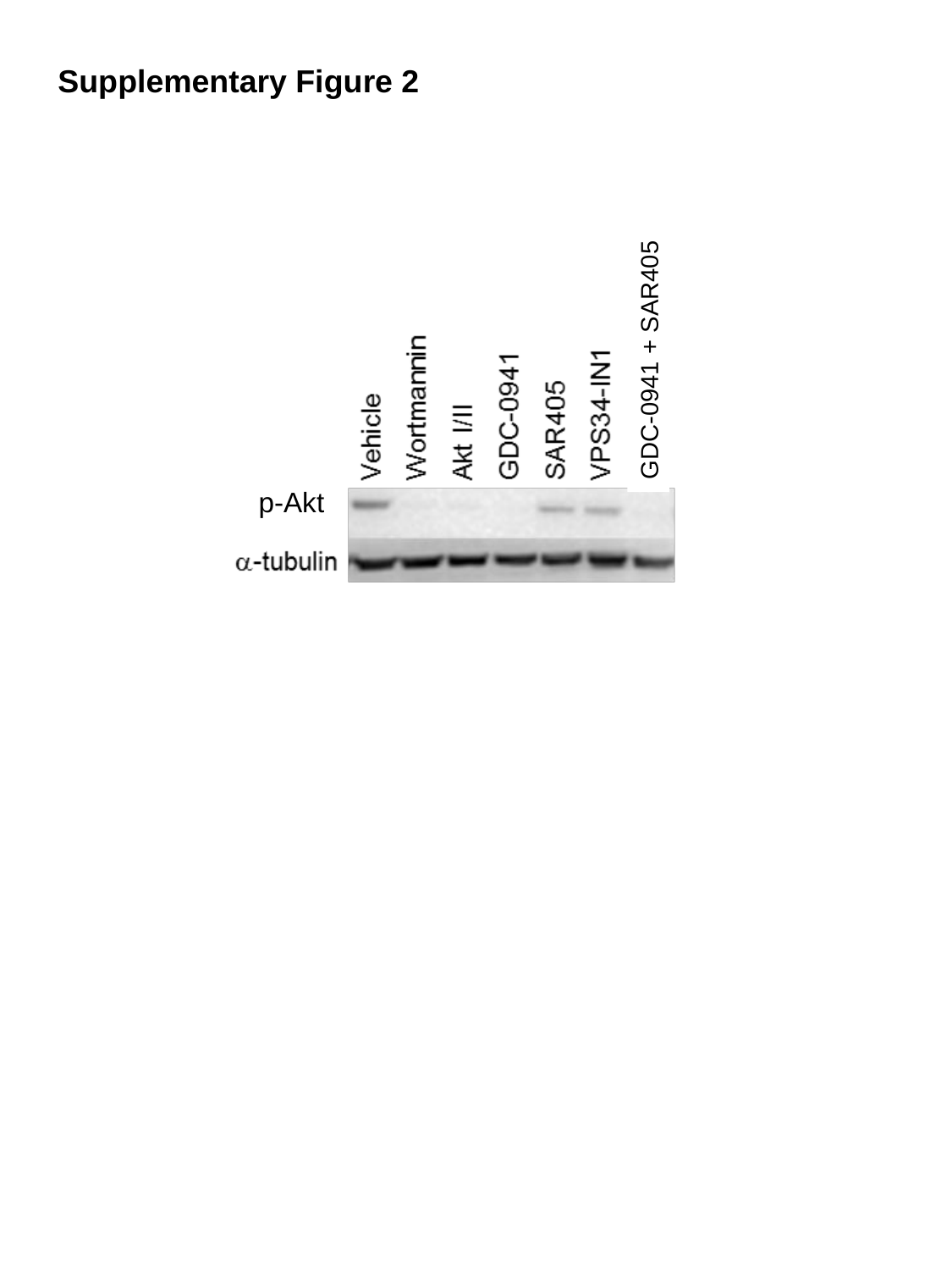

Supplementary Figure 2
GDC-0941 + SAR405
p-Akt

## Slide 3
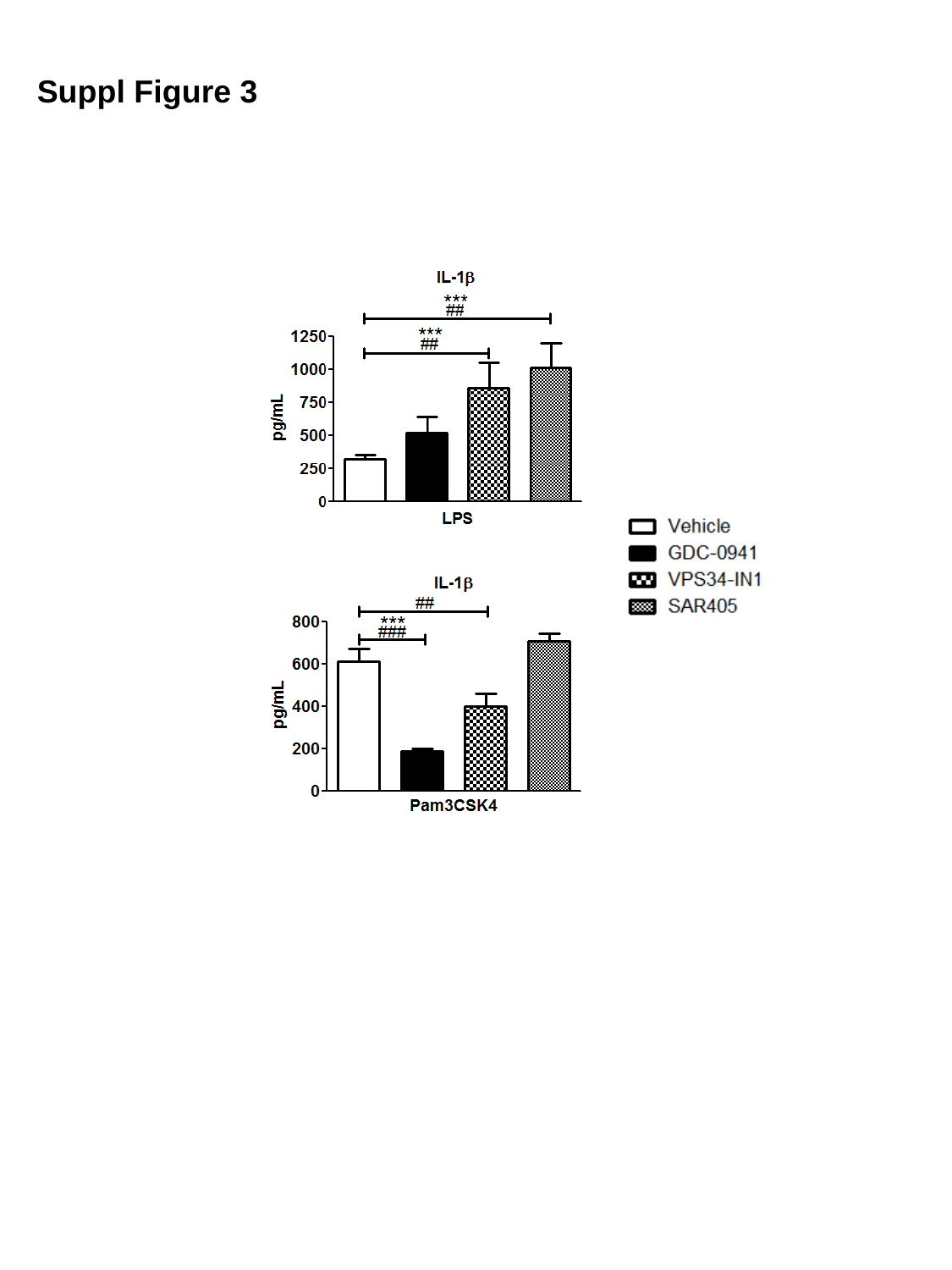

Suppl Figure 3
